# Reproducibility of pharmacogenetics findings for paclitaxel in a heterogeneous population of patients with lung cancer

**DOI:** 10.1371/journal.pone.0212097

**Published:** 2019-02-28

**Authors:** Tristan M. Sissung, Arun Rajan, Gideon M. Blumenthal, David J. Liewehr, Seth M. Steinberg, Arlene Berman, Giuseppe Giaccone, William D. Figg

**Affiliations:** 1 Clinical Pharmacology Program, Office of the Clinical Director, National Cancer Institute, Bethesda, Maryland, United States of America; 2 Thoracic and Gastrointestinal Oncology Branch, Office of the Clinical Director, National Cancer Institute, Bethesda, Maryland, United States of America; 3 Biostatistics and Data Management Section, Office of the Clinical Director, National Cancer Institute, Bethesda, Maryland, United States of America; 4 Office of Research Nursing in the Office of the Clinical Director, Office of the Clinical Director, National Cancer Institute, Bethesda, MD, United States of America; 5 Department of Oncology, Lombardi Comprehensive Cancer Center, Georgetown University, Washington, D.C., United States of America; National Cancer Center, JAPAN

## Abstract

Pharmacogenetics studies have identified several allelic variants with the potential to reduce toxicity and improve treatment outcome. The present study was designed to determine if such findings are reproducible in a heterogenous population of patients with lung cancer undergoing therapy with paclitaxel. We designed a prospective multi-institutional study that recruited *n* = 103 patients receiving paclitaxel therapy with a 5-year follow up. All patients were genotyped using the Drug Metabolizing Enzymes and Transporters (DMET) platform, which ascertains 1931 genotypes in 235 genes. Progression-free survival (PFS) of paclitaxel therapy and clinically-significant paclitaxel toxicities were classified and compared according to genotype. Initial screening revealed eleven variants that are associated with PFS. Of these, seven variants in *ABCB11* (rs4148768), *ABCC3* (rs1051640), *ABCG1* (rs1541290), *CYP8B1* (rs735320), *NR3C1* (rs6169), *FMO6P* (rs7889839), and *GSTM3* (rs7483) were associated with paclitaxel PFS in a multivariate analysis accounting for clinical covariates. Multivariate analysis revealed four SNPs in *VKORC1* (rs2884737), *SLC22A14* (rs4679028), *GSTA2* (rs6577), and *DCK* (rs4643786) were associated with paclitaxel toxicities. With the exception of a variant in *VKORC1*, the present study did not find the same genetic outcome associations of other published research on pharmacogenetics variants that affect paclitaxel outcomes. This finding suggests that prior pharmacogenomics research findings may not be reproduced in the most frequently-diagnosed malignancy, lung cancer.

## Introduction

Heritable variants in genes that affect drug absorption, distribution, metabolism, transport, and activation (ADME-A) may partly explain inter-individual differences in susceptibility to drug inefficacy or toxicity in lung cancer.[1–6] Such variants also play a role in the exposure to nicotine and nicotine dependence[7] and changes in drug metabolism that are induced by smoking. [8, 9]

Paclitaxel is approved as first-line therapy for patients with lung cancer. Hepatic uptake of paclitaxel is primarily regulated by the transporter OATP1B3 (encoded by *SLCO1B3*),[10] metabolized by several enzymes (e.g., CYP2C8 and CYP3A4),[11] and effluxed by ABCB1 (P-glycoprotein) and the bile acid transporters, ABCB4, and ABCB11.[12–14] Although controversial, several studies suggest some polymorphisms in these ADME genes are related to paclitaxel outcomes and toxicity.[15] Two studies have examined paclitaxel disposition and paclitaxel-induced neutropenia in a Dutch population (*N =* 279) using the DMET array–a platform that tests 1931 variants in 235 pharmacogenes.[2, 4] Yet, these studies did not find allelic variation in these genes was related to paclitaxel pharmacokinetics or toxicity, except for *ABCB11*.

The present study was designed to explore associations between ADME genes and paclitaxel outcome in patients undergoing various treatments in a multi-institutional setting, specifically focusing on whether previous pharmacogenomics findings would be validated in a clinically representative and non-uniform cohort taking taxanes as single agents and in combination with other therapies.

## Results

### Patients and treatment

Patient characteristics including age, race, gender, smoking history, and various chemotherapy treatment combinations are shown in Table 1. Most patients (*N =* 103) received either paclitaxel in combination with carboplatin (59%, *N =* 61), or paclitaxel and carboplatin in combination with other agents (33%, *N =* 34), with few receiving either paclitaxel alone (5.8%, *N =* 6), or paclitaxel combined with cisplatin (1.9%, *N =* 2). Clinical outcome measures included toxicities and progression-free survival (PFS). Given that hematological toxicity and neuropathy (*N =* 14) were the most frequently observed clinically significant toxicities, we chose to evaluate these outcomes. Other ≥ grade 3 toxicities were too infrequent (*N* ≤ 2) to evaluate associations with genotype. Overall, a total of *n* = 16 patients stopped paclitaxel therapy due to toxicity.

**Table 1:** Patient demographics, baseline disease characteristics, and treatment (safety analysis population).

	Carboplatin + Paclitaxel (N = 61)	Carboplatin + Paclitaxel and other agents (N = 34)	Cisplatin + Paclitaxel (N = 2)	Paclitaxel (N = 6)	Overall Total (N = 103)
**Age, y**					
Median	66.4	63.5	59.2	70.6	66.3
Minimum-Maximum	49.9–85.3	34.8–91.3	57.4–60.9	58.5–83.6	34.8–91.3
**Sex, N (%)**					
Male	43 (70)	14 (41)	1 (50)	2 (33)	60 (58)
Female	18 (30)	20 (59)	1 (50)	4 (67)	43 (42)
**Race, N (%)**					
White	36 (59)	24 (71)	2 (100)	6 (100)	68 (66)
Black	18 (30)	4 (12)	0 (0)	0 (0)	22 (21)
Asian	3 (4.9)	4 (12)	0 (0)	0 (0)	7 (6.8)
Hawaiian or Pacific Islander	1 (1.6)	0 (0)	0 (0)	0 (0)	1 (0.97)
Other	1 (1.6)	0 (0)	0 (0)	0 (0)	1 (0.97)
Not Specified	2 (3.3)	2 (5.9)	0 (0)	0 (0)	4 (3.9)
**Smoking classification, N (%); N missing = 1**					
Never smoked	10 (16)	8 (24)	0 (0)	0 (0)	18 (17)
Current smoker	6 (10)	2 (5.9)	0 (0)	0 (0)	8 (7.8)
Former smoker	44 (72)	24 (71)	2 (100)	6 (100)	76 (74)
**Non-metastatic vs metastatic, N (%)**					
Non-metastatic	37 (61)	10 (29)	0 (0)	0 (0)	47 (46)
Metastatic	22 (36)	21 (62)	2 (100)	2 (33)	47 (46)
N/A (Small Cell)	2 (3.3)	3 (8.8)	0 (0)	4 (67)	9 (8.7)
**Histology, N (%)**					
Adenocarcinoma	31 (51)	22 (65)	1 (50)	2 (33)	56 (54)
Bronchoalaveolar carcinoma	2 (3.3)	1 (2.9)	0 (0)	0 (0)	3 (2.9)
Squamous cell carcinoma	19 (31)	6 (18)	1 (50)	0 (0)	26 (25)
Small cell	2 (3.3)	3 (8.8)	0 (0)	4 (67)	9 (8.7)
Large cell carcinoma	1 (1.6)	1 (2.9)	0 (0)	0 (0)	2 (1.9)
Unclassified	6 (9.8)	1 (2.9)	0 (0)	0 (0)	7 (6.8)
**No. prior systemic therapy regimens, N (%)**					
0	48 (79)	22 (65)	2 (100)	1 (17)	73 (71)
1	9 (15)	7 (21)	0 (0)	1 (17)	17 (17)
2	2 (3.3)	4 (12)	0 (0)	1 (17)	7 (6.8)
3	0 (0)	0 (0)	0 (0)	1 (17)	1 (0.97)
4	0 (0)	1 (2.9)	0 (0)	2 (33)	3 (2.9)
5	2 (3.3)	0 (0)	0 (0)	0 (0)	2 (1.9)
**Other therapy in combination with paclitaxel, N (%)**					
Bevacizumab		17 (50)			
YM 155		8 (23.5)			
Cetuximab		2 (5.9)			
Irinotecan		2 (5.9)			
Gemcitabine		1 (2.9)			
Radiation		1 (2.9)			
Etoposide		1 (2.9)			
Bevacizumab + SSIP (i.e., antimesothelin)		1 (2.9)			
Cetuximab + Bevacizumab		1 (2.9)			
Pack-years smoked, N missing = 4					
Median	36.0 (N = 59)	20.5 (N = 32)	30.8	56.3	32.8 (N = 99)
Upper, lower quartile	15.0, 58.5	1.0, 45.6	Insuf. Data	Insuf. Data	8.0, 57.0
Second-hand smoke, N (%), N missing = 1					
Yes	52 (87)	27 (79)	2 (100)	5 (83)	86 (84)
No	8 (13)	7 (21)	0 (0)	1 (17)	16 (16)

### Genotype versus progression-free survival (PFS)

There were 72 variants for which the univariate Cox regression identified as *P*<0.05 whereas 44 variants were found potentially important after an exact trend test was applied S1 Table. Only eleven variants were considered for further consideration, having P<0.01 after combining into two categories (see last column in S1 Table). We also analyzed a set of potential covariates (race, histology/disease type, metastatic status, second hand tobacco, number of prior therapies, age, and cigarette pack-years, Table 1. Initial Cox proportional hazards regression results indicated that metastatic status was weakly associated with PFS and was excluded from further analyses (N = 94; OR (95% CI): 2.27 (0.711, 7.25)). Both stepwise and backward selection methods indicated that the number of prior therapies (0 vs. 1 vs. 2+; N = 73, 17, 13, respectively) was associated with PFS such that patients with one prior therapy are at less, or the same, risk of progression (HR (95%CI): 0.845 (0.281, 2.54)) as patients with no prior therapy; patients with two or more prior therapies are at more risk of progression (HR (95%CI): 2.43 (1.03, 5.72)) than those with no prior therapy Table 2. All other clinical and demographic parameters (i.e., race, histology, disease type, second hand smoke, age, and cigarette pack-years) were found to not be associated with PFS in univariate analyses and were not considered further. Thus, despite the heterogeneity of the patients included in the study, only the number of prior therapies would be worthy of consideration in a multivariable analysis of factors associated with PFS and in the interpretation of results

**Table 2:** Proportional hazards analysis of PFS (N = 99).

Variable Type	Parameter	Gene	Allele	Parameter Estimate	SE	Pr > ChiSq	HR	95% Hazard Ratio Confidence Limits	
SNP	rs4148768	*ABCB11*	*C>T*	1.673	0.539	0.002	5.33	1.85	15.3
	rs1051640	*ABCC3*	*A>G*	1.385	0.490	0.005	3.99	1.53	10.4
	rs1541290	*ABCG1*	*G>A*	-0.955	0.461	0.038	0.385	0.156	0.949
	rs735320	*CYP8B1*	*G>A*	1.797	0.480	0.0002	6.03	2.36	15.5
	rs7889839	*FMO6P*	*A>G*	1.244	0.438	0.005	3.47	1.47	8.19
	rs7483	*GSTM3*	*G>A*	-1.463	0.564	0.009	0.232	0.077	0.699
	rs6196	*NR3C1*	*T>C*	1.076	0.468	0.021	2.93	1.17	7.33
Covar.	Prior therapies: 0 vs. 1			-0.168	0.562	0.76	0.845	0.281	2.54
	Prior therapies: 0 vs. 2+			0.889	0.436	0.041	2.43	1.03	5.72

**Fig 1:**
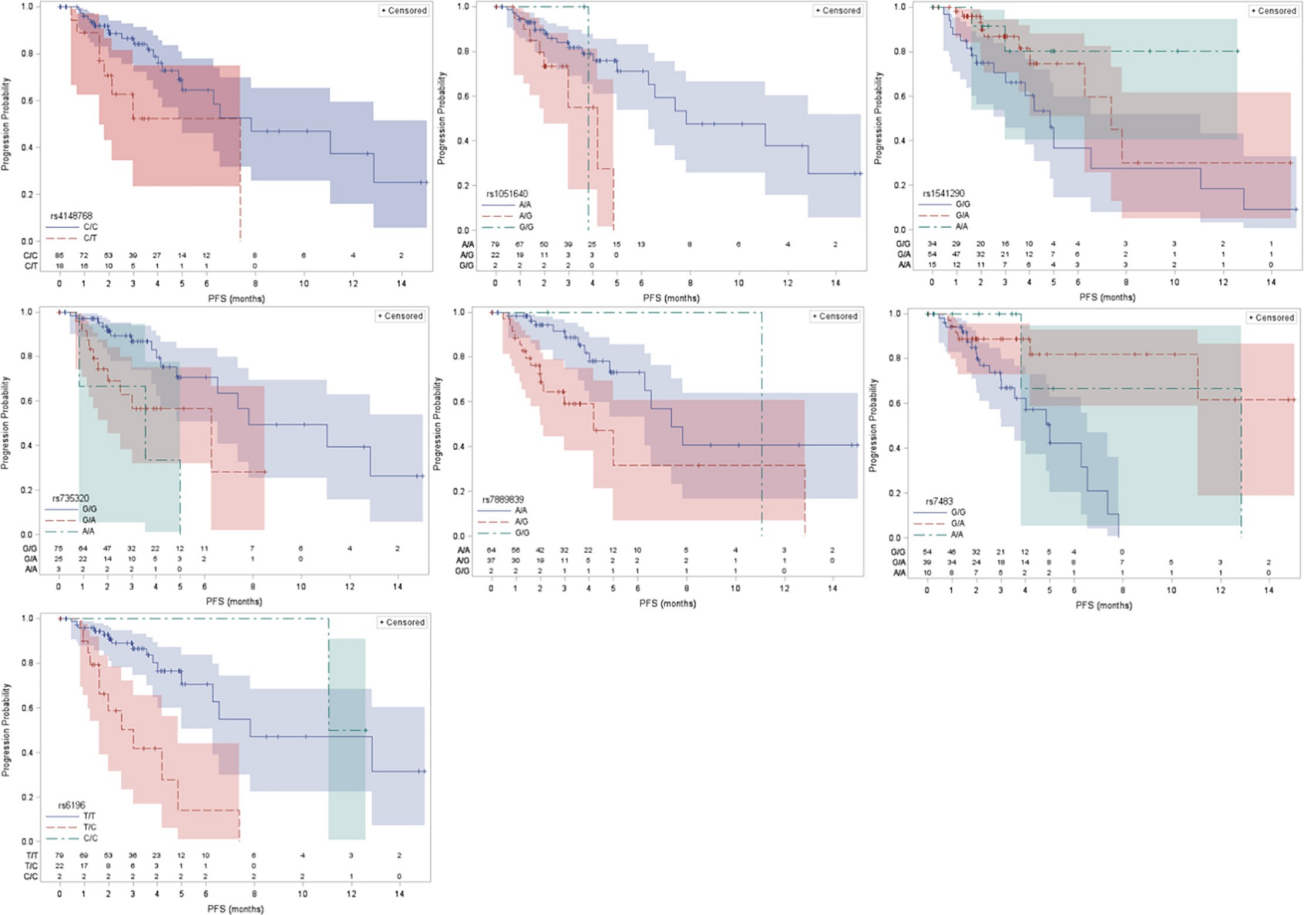
Kaplan-Meier plots of potentially important associations following Cox regression analyses. Paclitaxel PFS was related to genetic variants in seven genes: A) *ABCB11* rs4148768, B) *ABCC3* rs1051640, C) *ABCG1* rs1541290, *CYP8B1* rs735320, *FMO6P* rs7889839, *GSTM3* rs7483, and *NR3C1* rs6196.

Initial screening of the 12 SNPs using both backward and stepwise selection processes resulted in 7 SNPs in the final Cox model (both backward and stepwise selection processes agreed; see multivariable results including the 7 SNPs as well as number of prior therapies in Table 2. Remarkably, 5 of 7 potentially important genes identified in this study encode multiple enzymes and transporters involved in bile acid synthesis, canalicular transport of bile, and sterol clearance: *ABCB11* (Bile Salt Export Pump; BSEP), *ABCC3* (Canalicular Multispecific Organic Anion Transporter 2; CMOAT2), *ABCG1* (ATP-Binding Cassette Transporter G1), *CYP8B1* (Sterol 12-Alpha-Hydroxylase), and *NR3C1* (Glucocorticoid Receptor; GR). Other genes include a pseudogene, *FMO6P* (Flavin Containing Monooxygenase 6 Pseudogene), and the detoxification of electrophilic compounds, *GSTM3* (Glutathione S-Transferase, Mu-3). Median PFS and their 95% confidence intervals are provided in Table 3 and Kaplan-Meier plots are included in Fig 1.

**Table 3:** Median progression-free survival.

Gene	Variant	Stratum 1 (months)	Stratum 2 (months)
*ABCB11*	rs4148768	7.83 (5.00, NR*)	7.36 (1.31, 7.36)
*ABCC3*	rs1051640	7.83 (6.28, NR)	3.81 (2.99, 4.83)
*ABCG1*	rs1541290	4.83 (3.03, 11.08)	7.83 (6.28, NR)
*CYP8B1*	rs735320	7.83 (6.54, NR)	5.00 (2.01, NR)
*FMO6P*	rs7889839	7.36 (6.28, NR)	5.00 (2.14, 12.85)
*GSTM3*	rs7483	5.00 (3.03, 6.54)	12.85 (11.08, NR)
*NR3C1*	rs6196	7.83 (6.28, NR)	3.03 (1.61, 7.36)

* Not reached (NR)

### Genotype versus toxicity

Univariate screening of the 1931 SNPs indicated that only 46 had P<0.05 (S2 Table; both 2 x 2 and 2 x 3 cross-tabs) but only one of the seven 2 x 2 tables had P<0.01 (rs4679028; P = 0.004; OR (95% CI): 9.43 (1.68, 53.5)). Of the remaining 39 2 x 3 cross-tabs only 27 had sufficient observations for testing for a trend; the balance (12) were further analyzed after combining groups (see below). Of the 27 2 x 3 cross-tabs with sufficient data, only 5 had P<0.01 (rs4643786, rs910795, rs6811453, rs6577, rs1056836); Somers’ D indicates that the associations are most likely weak. Of the remaining 12 SNPs, only two had P<0.01 (rs3093105 (N = 85), rs2884737).

None of the covariates evaluated were considered in the multiple logistic regression analysis as they were found to be weakly associated with toxicity in the univariate analyses (race, histology/disease type, metastatic status, second hand tobacco, number of prior therapies, age, and cigarette pack-years (S3 Table). An initial analysis indicated that the variant corresponding to rs3093105 was not an important predictor of toxicity, so it was dropped from further consideration in analyses. Two similar, but competing, models were identified. In both models, variants in the vitamin K epoxide reductase complex (*VKORC1*; rs2884737) and the organic-cation transporter-like 4 (*OCTL4*, *SLC22A14*; rs4679028) were included. The odds ratios (OR; 95% CI) were 0.092 (0.002, 0.807) and 9.77 (1.65, 63.8), respectively for VKORC1 and SLC22A14 in Model 1, while they were 0.097 (0.002, 0.909) and 5.60 (0.992, 33.1) in Model 2; Table 4). Two other variants only appeared in one model each. Variants in the glutathione S-transferase alpha-2 (*GSTA2*; rs6577) were only found in model 1 (OR (95%CI) = 6.03 (1.42, 28.9) and those in the deoxycytidine kinase (*DCK*; rs4643786) were found in model 2 OR (95% CI) = 0.111 (0.009, 0.899), respectively). Model 1 correctly predicted 72/86 (83.7%) of those without toxicity and 11/16 (68.8%) of those with toxicity, and Model 2 correctly predicted 82/87 (94.3%) of those without toxicity and 8/16 (50.0%) of those with toxicity. The former model provided a more balanced set of classification probabilities, and is considered preferable for that reason. Different associations were found when hematological toxicities were considered as a group, and neutropenia and thrombocytopenia were considered as individual categories (S4 Table).

**Table 4:** Logistic regression models of toxicity.

Gene	Genotype with Higher Risk	Parameter	Parameter Estimate	SE	OR	95% Odds Ratio Confidence Limits		P (Exact)
**Model 1 (*N =* 102)**								
* *		Intercept	-2.288	0.491				
*VKORC1*	*T/T*	rs2884737	-2.49	1.171	0.092	0.002	0.807	0.024
*GSTA2*	*A/C*, *C/C*	rs6577	1.864	0.673	6.03	1.42	28.9	0.012
*SLC22A14*	*A/A*	rs4679028	2.425	0.817	9.77	1.65	63.8	0.010
**Model 2 (*N =* 103)**								
* *		Intercept	0.592	0.949				
*VKORC1*	*T/T*	rs2884737	-2.414	1.235	0.097	0.002	0.909	0.037
*DCK*	*C/C*	rs4643786	-2.394	0.975	0.111	0.009	0.899	0.038
*SLC22A14*	*A/A*	rs4679028	1.845	0.781	5.60	0.992	33.1	0.051

## Conclusions

The present study identified 7 genetic variants that were associated with paclitaxel PFS and 3 variants associated with major paclitaxel-induced dose-limiting toxicities in patients with lung cancer treated with paclitaxel. Findings included few genes that have been previously associated with paclitaxel outcomes and/or lung cancer progression. Other genes represent novel associations as are summarized below. The present data suggest that pharmacogenomics associations demonstrated in previous studies are not replicated in a clinically representative cohort of patients with lung cancer who have undergone paclitaxel therapy.

A previous study by de Graan *et al*. discovered that a set of four linked polymorphisms in *VKORC1* was related to low paclitaxel clearance in a cohort of individuals with a variety of cancers.[2] In the present study, the *VKORC1* SNP associated with a higher probability of toxicity, rs2884737 (T/T), is in strong linkage disequilibrium with the same allele (T/T) in rs9934438 that was associated with low clearance S1 Fig. Taken together with the findings of de Graan et al., our data suggest that this haploblock is associated with low clearance, and therefore a high probability of paclitaxel toxicity. However, the mechanism underlying the relationship between these SNPs and clearance/toxicity is currently unclear since, to our knowledge, VKORC1 activity regulates the oxidation state of vitamin K and clotting factors, which appears to have little relationship with drug metabolism or activity.

Four of seven (57.1%) variants identified in association with PFS were associated with bile synthesizing and transporting proteins (*ABCB11*, *ABCC3*, *ABCG1*, and *CYP8B1*). Bile acid enzymes and transporters have been previously associated with paclitaxel and docetaxel neutropenia,[4, 16] and, as explained above, some of these genes have previously been associated with paclitaxel outcomes for other reasons. For instance, ABCB11 transfection confers a low level of paclitaxel resistance in ovarian carcinoma cells, and is likely involved in hepatobiliary paclitaxel elimination,[12] and paclitaxel is an inhibitor of the BSEP.[17] However, *ABCB11* rs2287622 identified in Nieuweboer *et al*. (4 or 31) is not in linkage with the presently associated polymorphism, rs4148768. We propose that bile disposition is responsible for changing the expression of various drug metabolizing enzymes and transporters that are controlled by bile-responsive nuclear receptors,[18] and this possibility has been very poorly explored in the literature. For example, cholic acid induces *ABCC3* expression, which provides a hepatoprotective effect during cholestasis,[19] but the promotion of ABCC3 expression would also be expected to have significant consequences on paclitaxel and platinum disposition.[20–28]

Other findings are potentially related to the combination of multiple therapeutics. ABCC3 was identified as one of the most up-regulated genes in in chemotherapy-resistant lung cancer[28] and taxane-resistant breast cancer[22]. In lung cancer, carboplatin is responsible for increasing the expression of MRP3,[23] and acquired paclitaxel resistance during carboplatin cotherapy often appears to be a function of MRP3.[21] Many cisplatin-treated cells also upregulate MRP3,[20] and platinum resistance is associated with MRP3 in lung cancer.[26, 27] The polymorphism identified in the present study (rs1051640; E1503E) has also been observed in relation to cisplatin-induced ototoxicity;[24, 25] however, no study has yet characterized the functional effects of E1503E to our knowledge. DCK typically catalyzes the rate-limiting step of the formation of deoxynucleoside triphosphates, and the present SNP (rs4643786, 948T>C) is related to lower DCK expression in lymphocytes.[29] Consistent with this finding, 948C carriers with AML receiving topoisomerase inhibitors had superior response than those who carried 948TT [30]. DCK also phosphorylates gemcitabine, promoting its incorporation into DNA. Paclitaxel increases expression of dCK in NSCLC cell lines.[31] Our observations indicate that paclitaxel caused greater toxicity in patients carrying the low-DCK expressing genotype. This polymorphism should be studied further in this context.

Still other findings could be related to tumor progression. ABCG1 effluxes intracellular cholesterol to high-density lipoprotein particles that undergo reverse cholesterol transport from the periphery to the liver. Macrophages with ABCG1 expression undertake a tumor promoting phenotype, M2, and have been associated with tumor growth and antiapoptosis.[32] ABCG1 expression in macrophages is also associated with poor survival outcomes in patients with lung cancer.[33, 34] Accordingly, a polymorphism in *ABCG1* that was related to higher *ABCG1* expression (rs225388) was associated with poor survival in patients with lung cancer.[35] Another SNP (rs492338) was strongly associated with paclitaxel-induced neuropathy,[36] but this SNP was not associated with outcome in our study. Interestingly, the currently associated SNP (rs1541290 G/G) was previously related to low docetaxel clearance; which counterintuitively suggests that those carrying low clearance alleles had shorter PFS.[37] Therefore, it is unclear whether this polymorphism is predictive or prognostic, and could be both.

A polymorphism in the glucocorticoid receptor (*GR*; *NR3C1*) was also associated with poor PFS. Basal expression of CYP2C8 is regulated by the glucocorticoid receptor, and dexamethasone induces CYP2C8.[38, 39] Glucocorticoids reduce paclitaxel efficacy because GCs reduce paclitaxel-induced apoptosis, and blocking GR restores paclitaxel sensitivity.[40–43] High GR expression is related to LC cell survival[44] and corticosteroid co-therapy typically results in therapeutic resistance in lung cancer.[45] The N766N (rs6196 T>C) polymorphism in the glucocorticoid receptor is present in exon 9α, which comprises the ligand binding,[46] AP-1/NF-κB/dimerization/nuclear translocation domains of the *GR* and was associated with corticosteroid resistance in Crohn’s disease [47] although this finding is controversial.[48] Therefore, the glucocorticoid receptor could both affect paclitaxel metabolism and promote growth and prevent death signaling in lung cancers, and the mechanism behind the association of this polymorphism and poor paclitaxel PFS remains unclear.

Other findings include variants in GSTA2, GSTM3, FMO6P, and SLC22A14. GSTM3 is broadly distributed in the airway epithelium and smooth muscle of the lung and is expressed at higher levels in smokers than in ex-smokers.[49] Several GSTs have also been related to outcomes and clearance of paclitaxel.[2, 4] SNPs in GSTM3 and GSTA2 have not yet been explored in relation to paclitaxel outcomes in the literature.

With the exception of variants in *VKORC1*, most previously-studied genetic variants that are often related to paclitaxel pharmacokinetics and outcome were not reproducible in this study.[2, 4, 10–15] We suggest that such a finding would be expected since pharmacogenetics research is typically conducted retrospectively on clinical trials that select rather homogeneous populations of individuals in a controlled setting. By contrast, paclitaxel is most-often combined with a wide variety of agents in heterogenous populations with numerous different diseases in the community setting, Thus, prior pharmacogenomics findings are not reproducible in a prospectively recruited, clinically representative, and non-uniform cohort taking taxanes as single agents or in combination with other therapies. Such a finding is unfortunate for those undergoing paclitaxel therapy for the most frequently diagnosed malignancy, lung cancer. This study is based on a heterogeneous population and because of the limited number of patients which would be present in each population subgroup, it was not practical to further explore the associations of SNPs and outcomes in the subgroups. Except possibly for the number of previous systemic treatments, with the marginal association with PFS of the few patients with 2 or more prior treatments vs. those without any prior treatments, this was not likely to result in substantially different findings given that the various subgroups themselves were not found to differ in prognosis. However, it would be worthwhile if future evaluations on larger, possibly more homogeneous populations of patients could be undertaken to more definitively confirm the findings of the present study.

## Materials and methods

### Patients and treatment

A total of 546 patients with histologic diagnosis of primary lung carcinoma were enrolled in this study (NCT#00923884) between 2009 and 2012. Patients were treated at the National Cancer Institute (N = 441) and the Washington D.C. Veteran’s Affairs Medical Center (N = 105), and the present analysis focuses on those treated with paclitaxel. Of these 546 patients, 103 patients who received paclitaxel, either alone (N = 6) or in combination (N = 97), were successfully genotyped and had available smoking history, progression-free survival (PFS), and toxicity data. The study enrolled individuals who were over the age of 18 with non-small cell or small cell lung cancer who received any treatment (surgical resection, chemotherapy, radiation, or molecularly targeted therapy), had ECOG performance status of 0–3, and normal or impaired organ function. Those with a current diagnosis of or a prior history of other cancers were also enrolled. The study was approved by the Institutional Review Board at the National Cancer Institute (Bethesda) and the Veteran’s Affairs Medical Center (Washington D.C.). All patients provided written informed consent, and data for the present study are included on https://www.ncbi.nlm.nih.gov/gap.

### Assessments

All patients were followed for disease recurrence and survival for up to a 5-year period following enrollment. PFS was calculated by subtracting the date of initiation of a particular treatment from the date of progression on that treatment (censored if the patient was alive and remained on treatment as of the last follow-up). For the purposes of this analysis, only grade 3 or greater toxicities possibly, probably, or definitely related to the therapy were assessed. All patients received a questionnaire ascertaining cigarette pack years and secondhand tobacco exposure.

### Sample handling

A whole blood sample was obtained from each patient in an EDTA (lavender) top tube. DNA was extracted via ethanol precipitation, and samples were stored at -80°C until genotyping was conducted. Prior to genotyping, all samples underwent gDNA quantitation using the human RNase P TaqMan Copy Number Reference Assay (Thermo Fisher Scientific, Waltham, MA, USA) per the manufacturer’s instructions. Samples were diluted to 5ng/uL (150ng total) for analysis by the Drug Metabolizing Enzymes and Transporters platform (DMET; Affymetrix, Santa Clara, CA), which was also conducted using the manufacturer’s instructions. The DMET array ascertains 1931 allelic variants in 235 pharmacogenes. Of these 21% SNPs had at least one missing value, which is typically attributable to poor sample integrity or low DNA concentration; albeit, many other reasons are possible. All genotype and phenotype data used in this study are posted on dbGAP: http://www.ncbi.nlm.nih.gov/projects/gapprev/gap/cgi-bin/preview1.cgi?GAP_phs_code=PJfddSlJgReHqFfc

### Statistical considerations

Univariate genotype relationships with progression-free survival (PFS) were initially performed using a univariate proportional hazards (Cox) regression analysis and the Chi-square test to calculate the P-value as a screen. We next performed actuarial analysis on the PFS data and used both a log-rank based trend test and log-rank test to calculate two-tailed *P*-values. SNPs for which *P*<0.05 were noted for both Cox and actuarial analyses and were then further analyzed via Kaplan-Meier plots. If there were two strata, then a log-rank test was performed to compare stratum (exact tests were used if N<6 for either stratum). If there were three strata, and at least one stratum had N<6, then an exact trend test and log-rank test was performed. For those tests where P<0.05 Kaplan-Meier plots were produced and examined to determine whether adjacent stratum could be combined (e.g., G/A+A/A, but not G/G+A/A). If any of the strata were combined, then another log-rank test was performed. Finally, a multivariable Cox proportional hazards analysis including the SNPs identified by the univariate analyses, as well as any of the clinical or demographic factors identified to be potentially associated with PFS on univariate analyses, was performed to demonstrate the joint impact of the SNPs and any clinical or demographic factors on PFS.

Univariate genotype relationships with toxicity were first screened using Fisher’s exact test (2 x 2 tables) or Mehta’s modification to Fisher’s Exact test (2 x 3 tables), as appropriate [50]. Second, when applicable, if the two-tailed P-value from the first test was below 0.05, an exact Cochran-Armitage trend test was conducted. Finally, if a 2 x 3 table had small sample sizes (*N*<6) in any of the levels, adjacent levels were combined (e.g., 1+2, or 2+3) and then a Fisher’s exact test was conducted on the newly-created 2 x 2 table. If the last *P*-value was ≤0.010, then an odds ratio or Somers’ D was calculated (and 95% confidence intervals).

To further interpret the association between SNPs and toxicity, we also analyzed the SNPS along with a set of potential covariates (race, histology/disease type, metastatic status, second hand tobacco, number of prior therapies, age, and cigarette pack-years) in a multiple logistic regression analysis, which consisted of both backward and stepwise selection processes.

Given that this study is subject to a high number of comparisons, a *P*-value <0.010 was considered potentially important whereas *P*≤0.001 with sample sizes >30 in each group was considered to afford the most interpretive weight.

## Supporting information

S1 Fig The polymorphism identified in the present study, rs2884737, is in strong linkage with the haplotype block identified by de Graan et al.[2]. (PDF)

S1 Table Results of univariate screening of variants vs PFS. ^1^P-value calculated by the Chi-square test.
(PDF)

S2 Table Results of univariate screening of variants vs toxicity. ^1^Mehta’s modification to Fisher’s Exact test were conducted where appropriate. ^2^Combined categories with small sample sizes.
(PDF)

S3 Table Results of multiple logistic regression analysis of covariates. (PDF)

S4 Table Results of univariate screening of variants when neutropenia and thrombocytopenia were considered as individual categories. ^1^Mehta’s modification to Fisher’s Exact test were conducted where appropriate. ^2^Combined categories with small sample sizes.
(PDF)

## References

[1] Arbitrio M, Di Martino MT, Barbieri V, Agapito G, Guzzi PH, Botta C, et al Identification of polymorphic variants associated with erlotinib-related skin toxicity in advanced non-small cell lung cancer patients by DMET microarray analysis. Cancer Chemother Pharmacol. 2016;77(1):205–9. 10.1007/s00280-015-2916-3 . 26607259

[2] de Graan AJ, Elens L, Smid M, Martens JW, Sparreboom A, Nieuweboer AJ, et al A pharmacogenetic predictive model for paclitaxel clearance based on the DMET platform. Clin Cancer Res. 2013;19(18):5210–7. 10.1158/1078-0432.CCR-13-0487 . 23918604

[3] Drenberg CD, Paugh SW, Pounds SB, Shi L, Orwick SJ, Li L, et al Inherited variation in OATP1B1 is associated with treatment outcome in acute myeloid leukemia. Clin Pharmacol Ther. 2016;99(6):651–60. 10.1002/cpt.315 26663398 PMC4898266

[4] Nieuweboer AJ, Smid M, de Graan AJ, Elbouazzaoui S, de Bruijn P, Martens JW, et al Predicting paclitaxel-induced neutropenia using the DMET platform. Pharmacogenomics. 2015;16(11):1231–41. 10.2217/pgs.15.68 . 26265135

[5] Rumiato E, Boldrin E, Malacrida S, Battaglia G, Bocus P, Castoro C, et al A germline predictive signature of response to platinum chemotherapy in esophageal cancer. Transl Res. 2016;171:29–37 e1. 10.1016/j.trsl.2015.12.011 . 26772957

[6] Sissung TM, English BC, Venzon D, Figg WD, Deeken JF. Clinical pharmacology and pharmacogenetics in a genomics era: the DMET platform. Pharmacogenomics. 2010;11(1):89–103. 10.2217/pgs.09.154 . 20017675 PMC6448402

[7] Bergen AW, Michel M, Nishita D, Krasnow R, Javitz HS, Conneely KN, et al Drug Metabolizing Enzyme and Transporter Gene Variation, Nicotine Metabolism, Prospective Abstinence, and Cigarette Consumption. PLoS One. 2015;10(7):e0126113 10.1371/journal.pone.0126113 26132489 PMC4488893

[8] Do EK, Maes HH. Genotype x Environment Interaction in Smoking Behaviors: A Systematic Review. Nicotine Tob Res. 2016 10.1093/ntr/ntw153 . 27613915 PMC5896454

[9] Kroon LA. Drug interactions with smoking. Am J Health Syst Pharm. 2007;64(18):1917–21. 10.2146/ajhp060414 . 17823102

[10] Smith NF, Acharya MR, Desai N, Figg WD, Sparreboom A. Identification of OATP1B3 as a high-affinity hepatocellular transporter of paclitaxel. Cancer Biol Ther. 2005;4(8):815–8. . 16210916 10.4161/cbt.4.8.1867

[11] de Weger VA, Beijnen JH, Schellens JH. Cellular and clinical pharmacology of the taxanes docetaxel and paclitaxel—a review. Anticancer Drugs. 2014;25(5):488–94. 10.1097/CAD.0000000000000093 . 24637579

[12] Childs S, Yeh RL, Hui D, Ling V. Taxol resistance mediated by transfection of the liver-specific sister gene of P-glycoprotein. Cancer Res. 1998;58(18):4160–7. . 9751629

[13] Duan Z, Brakora KA, Seiden MV. Inhibition of ABCB1 (MDR1) and ABCB4 (MDR3) expression by small interfering RNA and reversal of paclitaxel resistance in human ovarian cancer cells. Mol Cancer Ther. 2004;3(7):833–8. . 15252144

[14] Januchowski R, Wojtowicz K, Andrzejewska M, Zabel M. Expression of MDR1 and MDR3 gene products in paclitaxel-, doxorubicin- and vincristine-resistant cell lines. Biomed Pharmacother. 2014;68(1):111–7. 10.1016/j.biopha.2013.09.004 . 24140176

[15] Frederiks CN, Lam SW, Guchelaar HJ, Boven E. Genetic polymorphisms and paclitaxel- or docetaxel-induced toxicities: A systematic review. Cancer Treat Rev. 2015;41(10):935–50. 10.1016/j.ctrv.2015.10.010 . 26585358

[16] Uchiyama T, Kanno H, Ishitani K, Fujii H, Ohta H, Matsui H, et al An SNP in CYP39A1 is associated with severe neutropenia induced by docetaxel. Cancer Chemother Pharmacol. 2012;69(6):1617–24. 10.1007/s00280-012-1872-4 . 22562553

[17] Wang EJ, Casciano CN, Clement RP, Johnson WW. Fluorescent substrates of sister-P-glycoprotein (BSEP) evaluated as markers of active transport and inhibition: evidence for contingent unequal binding sites. Pharm Res. 2003;20(4):537–44. . 12739759 10.1023/a:1023278211849

[18] Chiang JY. Bile acid regulation of hepatic physiology: III. Bile acids and nuclear receptors. Am J Physiol Gastrointest Liver Physiol. 2003;284(3):G349–56. 10.1152/ajpgi.00417.2002 . 12576301

[19] Teng S, Piquette-Miller M. Hepatoprotective role of PXR activation and MRP3 in cholic acid-induced cholestasis. Br J Pharmacol. 2007;151(3):367–76. 10.1038/sj.bjp.0707235 17435798 PMC2013976

[20] Kool M, de Haas M, Scheffer GL, Scheper RJ, van Eijk MJ, Juijn JA, et al Analysis of expression of cMOAT (MRP2), MRP3, MRP4, and MRP5, homologues of the multidrug resistance-associated protein gene (MRP1), in human cancer cell lines. Cancer Res. 1997;57(16):3537–47. . 9270026

[21] Melguizo C, Prados J, Luque R, Ortiz R, Caba O, Alvarez PJ, et al Modulation of MDR1 and MRP3 gene expression in lung cancer cells after paclitaxel and carboplatin exposure. Int J Mol Sci. 2012;13(12):16624–35. 10.3390/ijms131216624 23443122 PMC3546711

[22] O'Brien C, Cavet G, Pandita A, Hu X, Haydu L, Mohan S, et al Functional genomics identifies ABCC3 as a mediator of taxane resistance in HER2-amplified breast cancer. Cancer Res. 2008;68(13):5380–9. 10.1158/0008-5472.CAN-08-0234 . 18593940

[23] Oguri T, Isobe T, Fujitaka K, Ishikawa N, Kohno N. Association between expression of the MRP3 gene and exposure to platinum drugs in lung cancer. Int J Cancer. 2001;93(4):584–9. . 11477564 10.1002/ijc.1369

[24] Pussegoda K, Ross CJ, Visscher H, Yazdanpanah M, Brooks B, Rassekh SR, et al Replication of TPMT and ABCC3 genetic variants highly associated with cisplatin-induced hearing loss in children. Clin Pharmacol Ther. 2013;94(2):243–51. 10.1038/clpt.2013.80 23588304 PMC4006820

[25] Ross CJ, Katzov-Eckert H, Dube MP, Brooks B, Rassekh SR, Barhdadi A, et al Genetic variants in TPMT and COMT are associated with hearing loss in children receiving cisplatin chemotherapy. Nat Genet. 2009;41(12):1345–9. 10.1038/ng.478 . 19898482

[26] Young LC, Campling BG, Cole SP, Deeley RG, Gerlach JH. Multidrug resistance proteins MRP3, MRP1, and MRP2 in lung cancer: correlation of protein levels with drug response and messenger RNA levels. Clin Cancer Res. 2001;7(6):1798–804. . 11410522

[27] Young LC, Campling BG, Voskoglou-Nomikos T, Cole SP, Deeley RG, Gerlach JH. Expression of multidrug resistance protein-related genes in lung cancer: correlation with drug response. Clin Cancer Res. 1999;5(3):673–80. . 10100721

[28] Zhao Y, Lu H, Yan A, Yang Y, Meng Q, Sun L, et al ABCC3 as a marker for multidrug resistance in non-small cell lung cancer. Sci Rep. 2013;3:3120 10.1038/srep03120 24176985 PMC3814586

[29] Lamba JK, Crews K, Pounds S, Schuetz EG, Gresham J, Gandhi V, et al Pharmacogenetics of deoxycytidine kinase: identification and characterization of novel genetic variants. J Pharmacol Exp Ther. 2007;323(3):935–45. 10.1124/jpet.107.128595 . 17855478

[30] Falk IJ, Fyrberg A, Paul E, Nahi H, Hermanson M, Rosenquist R, et al Decreased survival in normal karyotype AML with single-nucleotide polymorphisms in genes encoding the AraC metabolizing enzymes cytidine deaminase and 5'-nucleotidase. Am J Hematol. 2013;88(12):1001–6. 10.1002/ajh.23549 . 23873772

[31] Shord SS, Patel SR. Paclitaxel alters the expression and specific activity of deoxycytidine kinase and cytidine deaminase in non-small cell lung cancer cell lines. J Exp Clin Cancer Res. 2009;28:76 10.1186/1756-9966-28-76 19500405 PMC2708129

[32] Sag D, Cekic C, Wu R, Linden J, Hedrick CC. The cholesterol transporter ABCG1 links cholesterol homeostasis and tumour immunity. Nat Commun. 2015;6:6354 10.1038/ncomms7354 25724068 PMC4347884

[33] Cardwell CR, Mc Menamin U, Hughes CM, Murray LJ. Statin use and survival from lung cancer: a population-based cohort study. Cancer Epidemiol Biomarkers Prev. 2015;24(5):833–41. 10.1158/1055-9965.EPI-15-0052 . 25934831

[34] Wong J, Quinn CM, Gelissen IC, Jessup W, Brown AJ. The effect of statins on ABCA1 and ABCG1 expression in human macrophages is influenced by cellular cholesterol levels and extent of differentiation. Atherosclerosis. 2008;196(1):180–9. 10.1016/j.atherosclerosis.2007.03.030 . 17466310

[35] Wang Y, Liu H, Ready NE, Su L, Wei Y, Christiani DC, et al Genetic variants in ABCG1 are associated with survival of nonsmall-cell lung cancer patients. Int J Cancer. 2016;138(11):2592–601. 10.1002/ijc.29991 26757251 PMC5294935

[36] Hertz DL, Roy S, Jack J, Motsinger-Reif AA, Drobish A, Clark LS, et al Genetic heterogeneity beyond CYP2C8*3 does not explain differential sensitivity to paclitaxel-induced neuropathy. Breast Cancer Res Treat. 2014;145(1):245–54. 10.1007/s10549-014-2910-1 24706167 PMC4256153

[37] Nieuweboer AJ, Smid M, de Graan AM, Elbouazzaoui S, de Bruijn P, Eskens FA, et al Role of genetic variation in docetaxel-induced neutropenia and pharmacokinetics. Pharmacogenomics J. 2016;16(6):519–24. 10.1038/tpj.2015.66 . 26345519

[38] Ferguson SS, Chen Y, LeCluyse EL, Negishi M, Goldstein JA. Human CYP2C8 is transcriptionally regulated by the nuclear receptors constitutive androstane receptor, pregnane X receptor, glucocorticoid receptor, and hepatic nuclear factor 4alpha. Mol Pharmacol. 2005;68(3):747–57. 10.1124/mol.105.013169 . 15933212

[39] Lai XS, Yang LP, Li XT, Liu JP, Zhou ZW, Zhou SF. Human CYP2C8: structure, substrate specificity, inhibitor selectivity, inducers and polymorphisms. Curr Drug Metab. 2009;10(9):1009–47. . 20214592 10.2174/138920009790711832

[40] Agyeman AS, Jun WJ, Proia DA, Kim CR, Skor MN, Kocherginsky M, et al Hsp90 Inhibition Results in Glucocorticoid Receptor Degradation in Association with Increased Sensitivity to Paclitaxel in Triple-Negative Breast Cancer. Horm Cancer. 2016;7(2):114–26. 10.1007/s12672-016-0251-8 26858237 PMC4789079

[41] Pang D, Kocherginsky M, Krausz T, Kim SY, Conzen SD. Dexamethasone decreases xenograft response to Paclitaxel through inhibition of tumor cell apoptosis. Cancer Biol Ther. 2006;5(8):933–40. . 16775428 10.4161/cbt.5.8.2875

[42] Reeder A, Attar M, Nazario L, Bathula C, Zhang A, Hochbaum D, et al Stress hormones reduce the efficacy of paclitaxel in triple negative breast cancer through induction of DNA damage. Br J Cancer. 2015;112(9):1461–70. 10.1038/bjc.2015.133 25880007 PMC4453678

[43] Wu W, Pew T, Zou M, Pang D, Conzen SD. Glucocorticoid receptor-induced MAPK phosphatase-1 (MPK-1) expression inhibits paclitaxel-associated MAPK activation and contributes to breast cancer cell survival. J Biol Chem. 2005;280(6):4117–24. 10.1074/jbc.M411200200 . 15590693

[44] Mihailidou C, Panagiotou C, Kiaris H, Kassi E, Moutsatsou P. Crosstalk between C/EBP homologous protein (CHOP) and glucocorticoid receptor in lung cancer. Mol Cell Endocrinol. 2016;436:211–23. 10.1016/j.mce.2016.08.001 . 27496643

[45] Taylor KM, Ray DW, Sommer P. Glucocorticoid receptors in lung cancer: new perspectives. J Endocrinol. 2016;229(1):R17–28. 10.1530/JOE-15-0496 . 26795718

[46] Tissing WJ, Meijerink JP, den Boer ML, Brinkhof B, van Rossum EF, van Wering ER, et al Genetic variations in the glucocorticoid receptor gene are not related to glucocorticoid resistance in childhood acute lymphoblastic leukemia. Clin Cancer Res. 2005;11(16):6050–6. 10.1158/1078-0432.CCR-04-2097 . 16115950

[47] Krupoves A, Mack D, Deslandres C, Seidman E, Amre DK. Variation in the glucocorticoid receptor gene (NR3C1) may be associated with corticosteroid dependency and resistance in children with Crohn's disease. Pharmacogenet Genomics. 2011;21(8):454–60. 10.1097/FPC.0b013e3283476a01 . 21633323

[48] Koper JW, Stolk RP, de Lange P, Huizenga NA, Molijn GJ, Pols HA, et al Lack of association between five polymorphisms in the human glucocorticoid receptor gene and glucocorticoid resistance. Hum Genet. 1997;99(5):663–8. . 9150737 10.1007/s004390050425

[49] Anttila S, Hirvonen A, Vainio H, Husgafvel-Pursiainen K, Hayes JD, Ketterer B. Immunohistochemical localization of glutathione S-transferases in human lung. Cancer Res. 1993;53(23):5643–8. . 8242618

[50] Mehta CR, Patel NR. A Network Algorithm for Performing Fisher Exact Test in R X C Contingency-Tables. J Am Stat Assoc. 1983;78(382):427–34. 10.2307/2288652 WOS:A1983QU74700037.

