# Supplementary figures and images for: Reproducibility of pharmacogenetics findings for paclitaxel in a heterogeneous population of patients with lung cancer
Source: PLoS One. 2019 Feb 28;14(2):e0212097. doi: 10.1371/journal.pone.0212097 (PMC6394902; doi:10.1371/journal.pone.0212097)

Figure S1

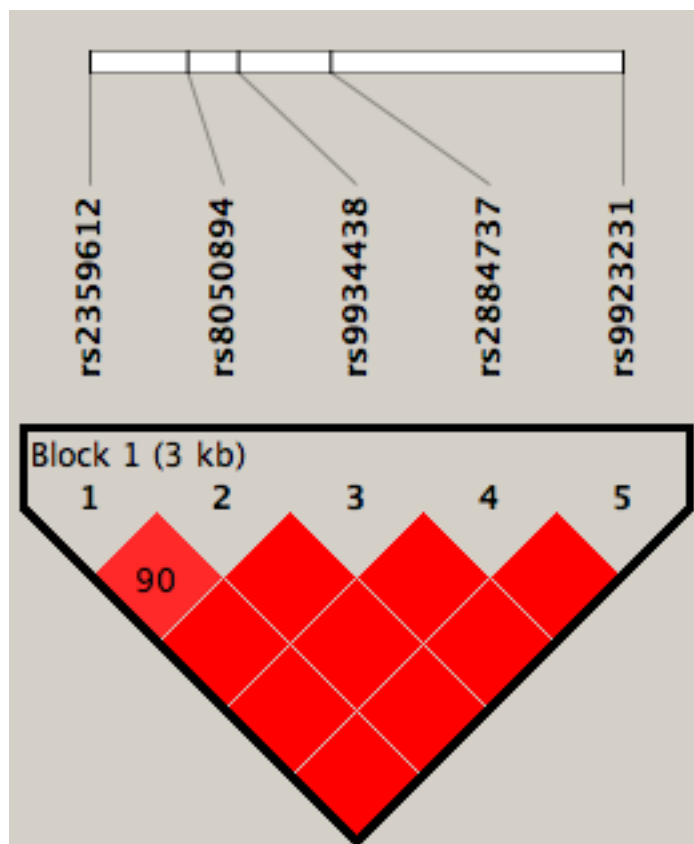

Supplement: S1 Fig — (PDF) [file pone.0212097.s001.pdf]
