# Supplementary material for: Reproducibility of pharmacogenetics findings for paclitaxel in a heterogeneous population of patients with lung cancer
Source: PLoS One. 2019 Feb 28;14(2):e0212097. doi: 10.1371/journal.pone.0212097 (PMC6394902; doi:10.1371/journal.pone.0212097)
Supplement: S1 Table — 1P-value calculated by the Chi-square test. (PDF) [file pone.0212097.s002.pdf]

Table S1. Results of univariate screening of variants vs PFS

SNP	Missing Genotypes *N*=	*n*<6 *N*=	Cox Regression[1] *P*=	Trend Test *P*=	Exact Trend Test *P*=	Log-rank Test *P*=
rs735320		y	**0.001**	**0.001**	**0.003**	**0.002**
rs171248			**0.002**	**0.002**	**0.006**	**0.003**
rs1789915			**0.002**	**0.002**	**0.007**	**0.010**
rs207440		y	**0.003**	**0.009**	0.035	**0.008**
rs149738			**0.005**	**0.004**	0.017	0.014
rs7483			**0.005**	**0.005**	**0.003**	**0.0004**
rs533486			**0.006**	**0.004**	0.017	0.014
rs16947			**0.009**	**0.007**	0.027	0.013
rs13959			**0.010**	**0.008**	0.028	0.018
rs1541290			0.011	**0.009**	0.025	**0.009**
rs1051640		y	0.021	0.037	0.11	**0.008**
rs7889839		y	0.032	0.017	0.018	**0.006**
rs7886938		y	0.032	0.017	0.018	**0.006**
rs2266780		y	0.045	0.041	0.034	0.014
rs279942			0.017	0.015	0.038	
rs183574			0.017	0.016	0.044	
rs6851610		y	0.019	0.029	0.096	
rs11584174		y	0.025	0.039	0.035	
rs8187755		y	0.029	0.066	0.13	
rs4149057			0.039	0.036	0.040	
rs6162			0.048	0.079	0.058	
rs1042640		y	0.036	0.033		0.025
rs17064		y	0.044	0.037		0.019
rs188096		y	0.047	0.039		0.038
rs2291075			0.017	0.015		
rs12179			0.025	0.023		
rs1080983			0.026	0.027		
rs1049793			0.032	0.029		
rs10929303			0.034	0.030		
rs1135840			0.034	0.031		
rs1800566			0.035	0.030		
rs1061040		y	0.035	0.031		
rs903247			0.037	0.033		
rs3756067			0.039	0.036		
rs11249460			0.041	0.036		
rs2302387			0.041	0.037		

rs12727968		y	0.044	0.039		
rs3788010			0.044	0.041		
rs17216275		y	**0.001**		0.048	
rs3862476			**0.002**		**0.003**	
rs4986782		y	**0.002**		0.057	
rs45446698		y	**0.005**		0.020	
rs67807361	45	y	**0.005**		0.088	
rs45494802		y	**0.006**		0.021	
rs3765534		y	**0.008**		0.16	
rs8191439		y	**0.009**		0.059	
rs4148768			**0.010**		**0.007**	
rs45467892	13	y	0.011		0.032	
rs67944833		y	0.011		0.058	
rs67944833		y	0.011		0.058	
rs57633837	40		0.013		0.010	
rs741817		y	0.013		0.012	
rs7798757		y	0.016		0.012	
rs7899457		y	0.016		0.039	
rs8187706		y	0.016		0.039	
rs1783811		y	0.016		0.049	
rs28399499		y	0.018		0.051	
rs8187838			0.022		0.016	
rs4148942		y	0.027		0.013	
rs5769		y	0.027		0.16	
rs915909		y	0.029		0.014	
rs41303343			0.030		0.019	
rs1051752		y	0.030		0.022	
rs7623741		y	0.030		0.27	
rs16975056		y	0.033		0.018	
rs10770865		y	0.033		0.13	
rs8177517		y	0.033		0.13	
rs9457846		y	0.033		0.13	
rs8187737		y	0.039		0.027	
rs4808326			0.039		0.033	
rs7334118		y	0.047		0.034	
rs28399454		y	0.049		0.019	
rs6196		y		0.019	**0.0002**	**0.001**
rs2032588		y		0.047	0.020	0.014
rs4149056		y		0.081	0.009	0.026
rs2762934		y		0.11	0.040	0.32

rs2470890			0.12	**0.006**	**0.002**
rs3731722		y	0.51	0.059	0.31
rs2053098			0.047	0.015	
rs55802895		y	0.22	0.059	
rs886205			0.44	**0.005**	
rs2276299		y	0.45	0.065	
rs2267665		y	0.045		0.048
rs1003973		y	0.047		0.049
rs2038067		y	0.047		0.049
rs2267669		y	0.047		0.049
rs2306283			0.047		
rs6980478		y		0.026	0.044
rs1229983		y		0.036	0.029
rs6163				0.015	
rs3770603		y		0.015	
rs6792261		y		0.017	
rs13119049				0.020	
rs13142440				0.021	
rs1064349	27	y		0.021	
rs17036104		y		0.021	
rs3731680		y		0.021	
rs998383				0.023	
rs2297809		y		0.023	
rs2644905		y		0.024	
rs4986907		y		0.024	
rs6193		y		0.024	
rs8187722		y		0.024	
rs1800822				0.026	
rs1517618		y		0.027	
rs10046				0.032	
rs1884725		y		0.034	
rs6965343				0.035	
rs1056892				0.038	
rs743572				0.038	
rs3748930				0.040	
rs700518				0.040	
rs4783745		y		0.041	
rs4148949		y		0.042	
rs55676788				0.044	
rs2277624				0.045	

rs4148951		0.046
rs7072219		0.046
rs3842	y	0.15
rs17863783	y	0.17
rs11678615	y	0.25

[1]P-value calculated by the Chi-square test.
