# Supplementary material for: Reproducibility of pharmacogenetics findings for paclitaxel in a heterogeneous population of patients with lung cancer
Source: PLoS One. 2019 Feb 28;14(2):e0212097. doi: 10.1371/journal.pone.0212097 (PMC6394902; doi:10.1371/journal.pone.0212097)
Supplement: S2 Table — 1Mehta’s modification to Fisher’s Exact test were conducted where appropriate. 2Combined categories with small sample sizes. (PDF) [file pone.0212097.s003.pdf]

Table S2. Results of univariate screening of variants vs toxicity

SNP rsID	Missing Genotypes *N*=	P1 OR (95% CI)	Fisher's Exact Test[1] *P*=	P2 Somers' D (95% CI)	Exact Trend Test *P*=	OR (95% CI)	Fisher's Exact Test[2] *P*=
Toxicity							
rs3093105	18		0.027		**0.009**	0.14 (0.01, 0.74)	**0.008**
rs2884737			**0.005**		0.053	0.10 (0.002, 0.75)	**0.010**
rs4643786			**0.007**	-0.16 (-0.34, 0.02)	**0.001**		
rs910795			0.013	0.19 (0.03, 0.36)	**0.005**		
rs6811453			0.024	-0.17 (-0.27, -0.06)	**0.008**		
rs6577			0.017	0.20 (0.02, 0.39)	**0.009**		
rs1056836			0.030	0.16 (0.05, 0.27)	**0.009**		
rs4679028		9.43 (1.68, 53.5)	**0.004**				
rs7081			0.014		0.012		0.020
rs2544794			0.021		0.009		0.022
rs2297810			0.044		0.020		0.027
rs1046428			0.027		0.013		0.065
rs7200749			0.040		0.027		0.066
rs1051741			0.032		0.032		0.066
rs894469			0.032		0.032		0.066
rs17878544			0.015		0.017		0.14
rs2266637			0.048		0.78		0.76
rs2738792			0.036		0.24		0.78
rs1056522			0.002		0.011		
rs11150564			0.027		0.013		
rs10002894			0.035		0.015		
rs11764079			0.033		0.016		
rs11770903			0.033		0.016		

rs1695		0.022	0.017
rs2277448		0.045	0.020
rs6830685		0.044	0.022
rs1826909		0.047	0.022
rs4715333		0.035	0.023
rs1056837		0.045	0.025
rs7957203		0.026	0.032
rs7867504		0.038	0.052
rs4926802	14	0.015	0.11
rs6987861		0.017	0.12
rs9934438		0.028	0.15
rs9923231		0.038	0.15
rs7294		0.041	0.15
rs8050894		0.031	0.22
rs2359612		0.038	0.29
rs2268873		0.020	0.83
rs2066853		0.050	0.83
rs2341968		0.011	
rs2550915		0.026	
rs1801030		0.029	
rs11568373		0.047	
rs1645691		0.047	
rs1051332		0.049	

[1]Mehta's modification to Fisher's Exact test were conducted where appropriate.

[2]Combined categories with small sample sizes.
