# Supplementary material for: Reproducibility of pharmacogenetics findings for paclitaxel in a heterogeneous population of patients with lung cancer
Source: PLoS One. 2019 Feb 28;14(2):e0212097. doi: 10.1371/journal.pone.0212097 (PMC6394902; doi:10.1371/journal.pone.0212097)
Supplement: S3 Table — (PDF) [file pone.0212097.s004.pdf]

Covariate	N	Strata	OR (95% CI)	P (Wald)
Race	99	White (68), Non-W (31)	1.91 (0.639, 5.72)	0.25
Histology	103	NSCLC (94), SCLC (9)	1.52 (0.177, 13.1)	0.69
Stage	94	M0 (47), M1 (47)	2.27 (0.711, 7.25)	0.16
Second Hand Tobacco	102	Yes (86), No (16)	1.30 (0.324, 5.19)	0.71
No. Prior Therapies	103	0 (73), >0 (30)	0.853 (0.275, 2.64)	0.78
Age	103	Continuous	0.991 (0.939, 1.05)	0.74
Cig. Pack Years	99	0 (18), >0 (81)	2.72 (0.449, 16.5)	0.27
