# Supplementary material for: Reproducibility of pharmacogenetics findings for paclitaxel in a heterogeneous population of patients with lung cancer
Source: PLoS One. 2019 Feb 28;14(2):e0212097. doi: 10.1371/journal.pone.0212097 (PMC6394902; doi:10.1371/journal.pone.0212097)
Supplement: S4 Table — 1Mehta’s modification to Fisher’s Exact test were conducted where appropriate. 2Combined categories with small sample sizes. (PDF) [file pone.0212097.s005.pdf]

Table S2. Results of univariate screening of variants vs toxicity

SNP rsID	Missing Genotypes *N*=	P1 OR (95% CI)	Fisher's Exact Test[1] *P*=	P2 Somers' D (95% CI)	Exact Trend Test *P*=	OR (95% CI)	Fisher's Exact Test[2] *P*=
Thromocytopenia							
rs1051741			**0.001**		**0.001**	16.8 (2.21, 131)	**0.002**
rs1046428			**0.001**		**0.002**	19.9 (2.16, 928)	**0.002**
rs6783962			**0.004**	-0.13 (-0.23, -0.04)	**0.003**		
rs7294			**0.006**	0.11 (0.01, 0.21)	**0.007**		
rs1056522			0.021	-0.13 (-0.21, -0.04)	**0.008**		
rs2227291			0.039	0.09 (0.005, 0.18)	**0.010**		
rs894469			0.009		0.009		0.026
rs3093105	18		0.048		0.025		0.027
rs2297810			0.031		0.062		0.040
rs17878544			0.024		0.020		0.091
rs10002894			0.017		0.013		
rs5952696			0.018		0.014		
rs7879356			0.018		0.014		
rs3856650			0.040		0.014		
rs6324			0.016		0.018		
rs2359612			0.041		0.018		
rs1883322			0.046		0.021		
rs7436962			0.046		0.024		
rs4643786			0.028		0.025		
rs6830685			0.046		0.031		
rs3742106			0.050		0.033		
rs2468110			0.026		0.035		

rs11764079		0.041		0.054		
rs11770903		0.041		0.054		
rs2295475		0.039		0.170		
rs7515157		0.035		0.240		
rs1799735		0.021		0.250		
rs4418728		0.044		0.440		
rs4986993		0.027		0.590		
rs3755740		0.040		0.660		
rs4803381		0.032		0.730		
rs11231825		0.035		0.820		
rs4679028		0.014				
rs67437265		0.023				
rs1051752		0.037				
rs1801030		0.042				
rs72551385		0.042				
Neutropenia						
rs2043449		**0.010**		0.020	9.52 (1.59, 66.1)	**0.005**
rs7747555		**0.003**		**0.003**	9.33 (1.42, 57.6)	**0.009**
rs6577		**0.004**	0.20 (0.04, 0.36)	**0.002**		
rs2180314		**0.005**	-0.13 (-0.23, -0.04)	**0.002**		
rs1695		**0.004**	0.13 (0.02, 0.24)	**0.003**		
rs2277448		0.012	-0.13 (-0.23, -0.04)	**0.004**		
rs4715354		0.019	-0.12 (-0.21, -0.02)	**0.009**		
rs910795		0.019	0.13 (-0.01, 0.27)	**0.010**		
rs4715333		0.020	-0.14 (-0.24, -0.04)	**0.010**		
rs1645691	18.2 (1.82, 164)	**0.006**				
rs1801030	9.44 (1.44, 58.3)	**0.009**				
rs2341968	13.5 (1.48, 103)	**0.010**				

rs9474342	0.011	0.063	0.015
rs11211402	0.029	0.027	0.023
rs1517618	0.015	0.012	0.024
rs1061040	0.012	**0.010**	0.029
rs2397118	0.017	0.017	0.035
rs1565814	0.039	0.069	0.039
rs1645694	0.038	0.033	0.045
rs7081	0.031	0.027	0.089
rs2738792	0.034	0.023	0.120
rs5085	0.043	0.054	0.430
rs1137115	0.002	0.740	0.470
rs1046428	0.020	0.081	0.680
rs2020869	0.021	0.150	0.680
rs11550605	0.037	1.000	0.680
rs10249788	0.004	0.750	0.710
rs4715332	0.028	0.019	
rs818202	0.043	0.022	
rs11684227	0.048	0.027	
rs11764079	0.017	0.035	
rs11770903	0.017	0.035	
rs7957203	0.001	0.180	
rs4926802	0.039	0.410	
rs939336	0.045	0.430	
rs2242046	0.027	0.610	
rs2268873	0.046	0.760	
rs2301157	0.003	1.000	
rs2066853	0.027	1.000	
rs2305367	0.049	1.000	
rs67944833	0.016		

rs67944833	0.016			
rs915909	0.030			
rs1059491	0.031			
rs2550915	0.048			
rs4407290	0.048			
rs28399499	0.049			
Any Hemotoxicity				
rs6577	**0.006**	0.23 (0.05, 0.41)	**0.003**	
rs910795	**0.009**	0.18 (0.02, 0.34)	**0.005**	
rs1695	**0.008**	0.14 (0.01, 0.27)	**0.007**	
rs2277448	0.015	-0.16 (-0.28, -0.04)	**0.007**	
rs2180314	0.022	-0.16 (-0.27, -0.05)	**0.009**	
rs28365062	0.020		0.027	0.016
rs1046428	0.007		0.004	0.019
rs7853758	0.043		0.045	0.031
rs2884737	0.010		0.120	0.031
rs11211402	0.045		0.047	0.032
rs2297810	0.032		0.015	0.038
rs2276707	0.048		0.049	0.041
rs7200749	0.025		0.017	0.042
rs1051741	0.020		0.020	0.042
rs894469	0.020		0.020	0.042
rs8023604	0.047		0.064	0.042
rs17882539	0.049		0.031	0.044
rs4643786	0.004		0.004	0.048

rs17878544	0.008	0.009	0.057
rs3805114	0.035	0.035	0.077
rs9474342	0.050	0.180	0.077
rs7747555	0.036	0.036	0.079
rs2268877	0.022	0.630	0.150
rs1137115	0.010	1.000	0.390
rs2738792	0.031	0.088	0.550
rs2268873	0.013	0.810	0.570
rs4803381	0.044	0.450	0.770
rs1060253	0.045	0.340	1.000
rs1051332	0.011	0.011	
rs11764079	0.027	0.011	
rs11770903	0.027	0.011	
rs5952696	0.026	0.019	
rs7879356	0.026	0.019	
rs1881668	0.044	0.021	
rs1056522	0.009	0.030	
rs4715333	0.033	0.030	
rs10002894	0.045	0.033	
rs1801249	0.029	0.036	
rs732774	0.032	0.036	
rs4926802	0.013	0.038	
rs2295475	0.029	0.039	
rs6830685	0.050	0.047	
rs3775770	0.026	0.076	
rs6783962	0.040	0.140	

rs1340990	0.040	0.300
rs2066853	0.039	0.490
rs1736565	0.040	0.660
rs4986993	0.022	1.000
rs2341968	0.006	
rs1801030	0.016	
rs28693844	0.018	
rs59443548	0.018	
rs16840132	0.018	
rs17215633	0.018	
rs2550915	0.018	
rs6921269	0.018	
rs4679028	0.019	
rs11568373	0.033	
rs1645691	0.033	
rs61743418	0.039	
rs72554635	0.039	
rs72554636	0.039	
rs2234934	0.039	
rs8187801	0.049	
rs12721607	0.049	
rs67944833	0.049	
rs67944833	0.049	
rs741817	0.050	
rs6577	0.006	
rs910795	0.009	

rs1695	0.008
rs2277448	0.015
rs2180314	0.022
rs28365062	0.020
rs1046428	0.007
rs7853758	0.043
rs2884737	0.010
rs11211402	0.045
rs2297810	0.032
rs2276707	0.048
rs7200749	0.025
rs1051741	0.020
rs894469	0.020
rs8023604	0.047
rs17882539	0.049
rs4643786	0.004
rs17878544	0.008
rs3805114	0.035
rs9474342	0.050
rs7747555	0.036
rs2268877	0.022
rs1137115	0.010
rs2738792	0.031
rs2268873	0.013
rs4803381	0.044
rs1060253	0.045

rs1051332	0.011
rs11764079	0.027
rs11770903	0.027
rs5952696	0.026
rs7879356	0.026
rs1881668	0.044
rs1056522	0.009
rs4715333	0.033
rs10002894	0.045
rs1801249	0.029
rs732774	0.032

[1]Mehta's modification to Fisher's Exact test were conducted where appropriate.

[2]Combined categories with small sample sizes.
